# Public availability of evidence-based mental health apps: a systematic review and meta-analysis of self-guided apps

**DOI:** 10.1038/s41746-026-03014-5

**Published:** 2026-07-16

**Authors:** Imogen H. Bell, Jake Linardon, Shaminka N. Mangelsdorf, Mario Alvarez-Jimenez, Lee Valentine, Emily Eisner, Kate A. Reynolds, John Torous, Inez Myin-Germeys, Sandra Bucci, David C. Mohr, Ulrich Reininghaus, Jennifer Nicholas

**Affiliations:** 1 Orygen, The National Centre of Excellence in Youth Mental Health, Melbourne, VIC Australia; 2 Centre for Youth Mental Health, University of Melbourne, Melbourne, VIC Australia; 3 School of Psychology, Faculty of Health, Deakin University, Geelong, VIC Australia; 4 Division of Psychology and Mental Health, University of Manchester, Manchester, UK; 5 Greater Manchester Mental Health NHS Foundation Trust, Manchester, UK; 6 Division of Digital Psychiatry, Beth Israel Deaconess Medical Center, Harvard Medical School, Boston, MA USA; 7 Centre for Contextual Psychiatry, KU Leuven, Leuven, Belgium; 8 Department of Preventive Medicine, Northwestern University, Chicago, IL USA; 9 Central Institute of Mental Health, University of Heidelberg, Mannheim, Germany; 10 Health Service and Population Research Department, Institute of Psychiatry, Psychology & Neuroscience, King’s College London, London, UK; 11 Centre for Society and Mental Health, King’s College London, London, UK; 12 German Center for Mental Health (DZPG) , partner site Mannheim-Heidelberg-Ulm, Mannheim, Germany

**Keywords:** Health care, Medical research, Psychology, Psychology

## Abstract

Despite mental health apps having a rigorous evidence base, little is known about the proportion of efficacious apps that are publicly available. Our systematic review and meta-analysis identified self-guided mental health apps evaluated in controlled trials, determined their public availability, and examined how evidence quality and strength influence availability. A literature search identified 16,971 records, of which 110 studies, investigating 112 apps, met inclusion criteria. Of the 81 unique self-guided apps, 42 (52%) were publicly available. Apps were most commonly available through both Apple App and Google Play Stores (79%) at no cost (40%). Study quality assessed using Cochrane Risk of Bias 2 tool had no significant association with app availability (*p* = 0.851). Meta-analysis found no significant difference (*p* = 0.228) in effect size compared to control between available apps (g = 0.33) and unavailable apps (g = 0.45). Considerations apart from evidence quality or strength determine app translation highlighting a gap between evidence and availability.

## Introduction

Mental health disorders are a leading contributor to global health-related burden, with recent evidence of an upward trajectory representing an escalation of mental health challenges worldwide^1^. In high-income countries, service capacity cannot keep pace with increasing demand, and with an estimated 82% of burden experienced in lower income countries, digital mental health interventions have emerged as a solution^2^. Indeed, a key tenant that has fuelled digital mental health research, innovation, and investment has been the potential for broadening access to mental health treatment and support. Able to transcend geographic boundaries, provide 24-hour access, and be anonymously accessible, smartphone mental health apps address many long-standing barriers to traditional mental health care.

Now spanning 20 years, digital mental health interventions generally, and apps specifically, have accumulated a substantial evidence base of efficacy across a range of conditions, populations, and therapeutic modalities^3,4^. Several recent meta-analyses have found apps efficacious for a range of mental health problems, including depression, anxiety, PTSD, and eating disorders, as well as transdiagnostically^2–5^. As this evidence base has matured, the field is shifting from establishing efficacy to examining the real-world implementation and dissemination of efficacious digital mental health tools^2,6^. However, like the health field more broadly, ensuring evidence-based interventions are available to those who could benefit remains an intractable and persistent challenge^7^.

Somewhat unique to digital health, translation barriers do not equate to a lack of mental health app options available to consumers. On the contrary, consumers face an abundance of choice. Over 10,000 mental health apps^8^, many self-guided, can be downloaded through commercial app stores. However, unlike the robust research literature, the vast majority of these available apps have not been evaluated in clinical trials, with audits suggesting that only a very small fraction have had their efficacy rigorously tested^9,10^. Consistent findings over 10 years of app research^11–13^ indicate that, overall, available apps are not backed by scientific evidence, do not adequately protect data privacy and security^14^, couch claims to side-step regulatory hurdles^9^, and include content that is not evidence-based and may, in some cases, be harmful^10,11,15^. The discrepancy between claims made by available mental health apps and their supporting empirical evidence has raised concerns about the quality and credibility of mental health apps available to consumers.

Less is known about the availability of self-guided apps that have undergone rigorous evaluation. Apps with an established evidence base may not be readily accessible through conventional channels such as app stores, which are the primary means by which users discover and download these digital tools. For example, a 2022 review of apps for people with a diagnosis of schizophrenia found that while just under half (48%) were available to the public, only 15% could be easily accessed through public marketplaces without requiring a code or special credentials^16^. This potential mismatch between the research evidence base and real-world availability highlights an important translational gap in understanding which evaluated digital mental health interventions ultimately reach consumers. Addressing this gap could support app selection by consumers and clinicians, while providing critical information to health systems, policy makers, and funders to inform app integration, policy and regulation, investment, and recommendation decisions.

Evidence quality has long guided decisions regarding the introduction and recommendation of new treatments^17^. Two factors by which evidence quality is commonly assessed, risk of bias and effect size, may, therefore, help elucidate whether evidence quality influences which apps reach consumers. Thus, the current systematic review and meta-analysis sought to explore the relationship between evidence quality and availability by: (i) identifying self-guided mental health apps evaluated in controlled trials; (ii) determining the availability of trialled self-guided mental health apps; and (iii) examining whether evidence quality and effect magnitude are related to app availability.

## Results

**Fig. 1:**
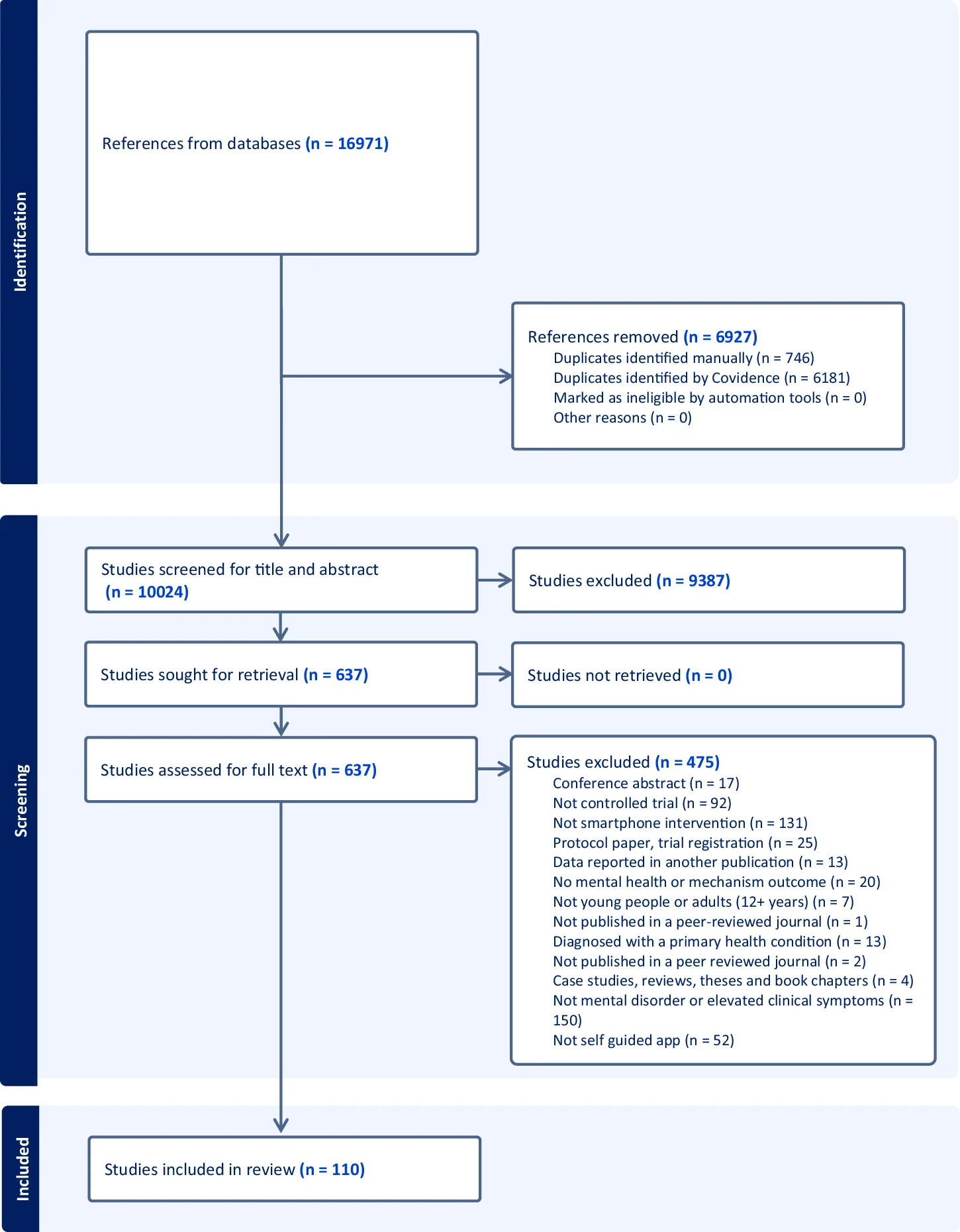
PRISMA flow diagram.

The search identified 16,971 records, of which 6927 were removed as duplicates. Following title and abstract screening, 637 articles proceeded to full text review. Of these, 110 met the inclusion criteria for the systematic review, and 96 reported sufficient pre and post data for inclusion in the meta-analysis. The PRISMA 2020 flow diagram outlining the selection process is presented in Fig. 1.

### Study and sample characteristics

The characteristics of each included study are detailed in Table 1. Across studies, sample sizes ranged from 15 to 4052, with a total pooled sample of 20,806 individuals (14,623 included in meta-analysis). Participants were predominantly adults (aged 26 years and older; 80 studies; 72.7%), with 30 studies (27.3%) focusing specifically on young people (aged 12–25). Most samples included individuals with subclinical or elevated symptom levels (73 studies; 66%), with the remainder including those with a clinical diagnosis of a mental health disorder (35 studies; 32%) or combining both clinical and subclinical groups (2 studies; 2%).

**Table 1:** Characteristics of included studies, app availability, effect sizes, and risk of bias

Author, year	App name	Country of origin	Population	*n*	Target condition	Intervention type	Control type	App availability	Availability location	Cost	Effect Size (Hedges’ *g*)	Risk of Bias
Abbott 2023	Headspace	USA	Young people (subclinical)	163	Anxiety disorder	Mindfulness-based	Passive	Available	Both app stores	Freemium/ subscription-based	0.135	!
Akin-Sari 2022	GG OCD (Turkish translation)	Turkey	Adults (subclinical)	55	Obsessive-compulsive disorder	CBT	Passive	Available	Both app stores	Freemium	0.797	!
Bantjes 2024 app 1	SuperBetter	South Africa	Young people (subclinical)	371	Mixed (transdiagnostic)	CBT	Active	Available	Both app stores	Subscription-based	0.333	!
Bantjes 2024 app 2	MoodFlow	South Africa	Young people (subclinical)	371	Mixed (transdiagnostic)	Multimodal/other	Active	Available	Both app stores	Free	0.241	!
Bell 2023	Mello	Australia	Young people (subclinical)	55	Mixed (transdiagnostic)	Transdiagnostic/mechanism-focused	Passive	Available	Both app stores	Free	0.409	-
Berman 2020*	TeleCoach	Sweden	Adults (subclinical)	89	Substance use disorder	Multimodal/other	Active	Unable to determine	Unable to determine	Unable to determine	-	!
Birney 2016*	Moodhacker®	USA	Adults (subclinical)	300	Depressive disorder	CBT+	Active	Available	Both app stores	Subscription-absed	-	!
Blanco 2022	CBIA	Spain	Adults (subclinical)	87	Depressive disorder	CBT	Active	Unable to determine	Unable to determine	Unable to determine	0.64	+
Boettcher 2018	Challenger	Sweden	Adults (clinical)	209	Anxiety disorder	CBT+	Passive	Unable to determine	Unable to determine	Unable to determine	.74 .58	!
Bovy 2022*	General app used to access intervention (e.g. EMA or WhatsApp)	Netherlands	Young people (subclinical)	96	Depressive disorder	Transdiagnostic/mechanism-focused	Active and passive	Not available	N/A	N/A	-	-
Bruhns 2021	MCT & More	Germany	Young people (subclinical)	423	Depressive disorder	Multimodal/other	Passive	Available	Both app stores	free	0.00	!
Bruhns 2023	MCT & More (renamed COGITO)	Germany	Adults (clinical)	159	Depressive disorder	Transdiagnostic	Passive	Available	Both app stores	Free	0.18	!
Bucci 2018	Actissist (Available as CareLoop)^	UK	Young people (clinical)	36	Schizophrenia spectrum disorder	CBT+	Active	Available	Both app stores	Free with access code	0.54	+
Bucci 2024	Actissist (Available as CareLoop)^	UK	Adults (clinical)	172	Schizophrenia spectrum disorder	CBT	Active	Available	Both app stores	Free with access code	0.27	+
Cardi 2022	FoodT	Italy	Adults (subclinical)	94	Feeding/eating/body image disorder	Transdiagnostic/mechanism-focused	Passive	Available	Both app stores	Free	0.42	!
Carl 2020	Daylight	UK	Adults (clinical)	256	Anxiety disorder	CBT	Passive	Available	Both app stores	Free with access code	0.68	!
Catuara-Solarz 2022	Foundations	UK	Adults (subclinical)	190	Anxiety disorder	Multimodal/other	Passive	Available	Both app stores	Free with access code	0.02	-
Cerea 2020	GG relationship Doubts (GGRO)	Italy	Young people (subclinical)	50	Obsessive-compulsive disorder	CBT	Passive	Available	Both app stores	Free	0.52	!
Cerea 2021	GG positive body image (GGBI)	Italy	Young people (subclinical)	50	Feeding/eating/body image disorder	CBT	Passive	Available	Both app stores	Freemium	0.59	!
Cerea 2022	GG OCD, anxiety, mood and sleep	Italy	Young people (subclinical)	95	Feeding/eating/body image disorder	CBT	Passive	Available	Both app stores	Freemium	0.61	!
Chan 2021	proACT-S	Hong Kong	Adults (clinical)	320	Mixed (transdiagnostic)	CBT	Passive	Unable to determine	Unable to determine	Unable to determine	0.85	!
Chun-Hung 2023*	Not determined	Taiwan	Adults (clinical)	99	Substance use disorder	Mindfulness-based	Active	Unable to determine	Unable to determine	Unable to determine	-	!
Dag 2014	General app used to access intervention (e.g. EMA or WhatsApp)	Sweden	Adults (clinical)	52	Anxiety disorder	CBT	Active	Not available	N/A	N/A	0.63	!
Dahne 2019a	Moodivate	USA	Adults (subclinical)	52	Depressive disorder	Behavioural activation	Active and passive	Available	Both app stores	Subscription-based	0.04.1.15	!
Dahne 2019b	¡Aptívate!	USA	Adults (subclinical)	42	Depressive disorder	Behavioural activation	Active and Passive	Unable to determine	Unable to determine	Unable to determine	0.08 0.36	!
Dahne 2023*	Goal2Quit	USA	Adults (subclinical)	164	Depressive disorder	Behavioural activation	Active	Not available	N/A	N/A	-	!
Danaher 2022	MomMoodBooster2	USA	Adults (subclinical)	191	Depressive disorder	CBT	Passive	Available	Website	Free with access code	0.33	!
Depp 2015	General app used to access intervention (e.g. EMA or WhatsApp)	USA	Adults (clinical)	104	Bipolar disorder	Psychoeducation	Active	Not available	N/A	N/A	−0.16	!
Donker 2019	ZeroPhobia	Netherlands	Adults (subclinical)	193	Anxiety disorder	CBT	Passive	Available	Both app stores	One time purchase	0.97	+
Donker 2023	ZeroPhobia vliegangst	Netherlands	Adults (subclinical)	154	Anxiety disorder	CBT	Passive	Available	Both app stores	One time purchase	1.16	-
Ebenfeld 2021	HelloBetter Panic	Germany	Adults (subclinical)	92	Anxiety disorder	CBT	Passive	Available	Both app stores	Free with Access Code	0.67	+
Ejiri 2023	Not Determined	Japan	Young people (subclinical)	32	Depressive disorder	Multimodal/other	Active	Unable to determine	Unable to determine	Unable to determine	−0.48	!
Farren 2021	UControlDrink	Ireland	Adults (clinical)	111	Substance use disorder	CBT	Passive	Not available	N/A	N/A	−0.32	!
Frank 2022*	Cue	USA	Adults (clinical)	135	Mixed (transdiagnostic)	Multimodal/other	Active	Not available	N/A	N/A	-	-
Gao 2022	Unwinding Anxiety®	USA	Adults (subclinical)	80	Mixed (transdiagnostic)	Mindfulness-based	Passive	Available	Both app stores	Subscription-Based	0.77	!
Gaudiano 2024	Mobile After-Care Support App	USA	Adults (clinical)	42	Schizophrenia spectrum disorder	CBT	Active	Not available	N/A	N/A	−0.28	-
Ghaemi 2022*	PEAR-004	USA	Adults (clinical)	112	Schizophrenia spectrum disorder	Multimodal/other	Active	Not available	N/A	N/A	-	!
Ghosh 2023*	TreadWill	India	Young people (subclinical)	598	Depressive disorder	CBT	Active and passive	Unable to determine	Unable to determine	Unable to determine	-	-
Harrer 2021	StudiCare Fernstudierende	Germany	Adults (subclinical)	200	Depressive disorder	Multimodal/other	Active	Not available	N/A	N/A	0.36	!
He 2022	XiaoE	China	Young people (subclinical)	148	Depressive disorder	CBT	Active and active	Unable to determine	Unable to determine	Unable to determine	1.27 .54	!
Hensler 2022	PTSD Coach	Sweden	Adults (subclinical)	179	Trauma-related disorder	Multimodal/other	Passive	Available	Both app stores	Free	0.52	!
Hinterberger 2023	Sleep2 sleeptracker & analysis, delveoped by NUKKUAA	Austria	Adults (subclinical)	59	Sleep disorder	CBT	Passive	Available	Google Play Store	Freemium	0.70	!
Horsch 2017	Sleepcare	Netherlands	Adults (clinical)	151	Sleep disorder	CBT	Passive	Unable to determine	Unable to determine	Unable to determine	0.70	!
Huberty 2021	Calm	USA	Adults (subclinical)	263	Sleep disorder	Mindfulness-based	Passive	Available	Both app stores	Subscription-based	0.05	-
Hur 2018	Todac Todac	South Korea	Young people (clinical)	48	Depressive disorder	CBT	Active	Available	Both app stores	Free	0.66	!
Ivanova 2016	Ångesthjälpen (The Anxiety Help)	Sweden	Adults (clinical)	152	Anxiety disorder	ACT	Passive	Unable to determine	Unable to determine	Unable to determine	0.27	!
Jannati 2020	Happy Mom	Iran	Adults (clinical)	78	Depressive disorder	CBT	Passive	Not available	N/A	N/A	2.95	!
Jongeneel 2024	Temstem	Netherlands	Adults (clinical)	89	Mixed (transdiagnostic)	Multimodal/other	Active	Available	Google Play Store	Free	−0.29	!
Kageyama 2021	Subliminal Priming with Supraliminal Reward Stimulation (SPSRS)	Japan	Young people (subclinical)	32	Depressive disorder	Multimodal/other	Passive	Unable to determine	Unable to determine	Unable to determine	0.25	!
Keeler 2022	FoodT	UK	Adults (clinical)	80	Feeding/eating/body image disorder	Multimodal/other	Passive	Available	Both app stores	Free	−0.12	!
Kennard 2018	Brite App	USA	Adolescents (subclinical)	66	Mixed (transdiagnostic)	Behavioural activation	Passive	Available	Apple App Store	Free with access code	−0.12	!
Kerber 2023	MindDoc	Germany	Adults (subclinical)	1045	Mixed (transdiagnostic)	Transdiagnostic/mechanism-focused	Passive	Available	Both app stores	Subscription-based	0.38	!
Kim 2024	Not Determined	South Korea	Adults (clinical)	50	Anxiety disorder	CBT	Active	Unable to determine	Unable to determine	Unable to determine	0.42	!
Krzystanek 2019	The MONEO platform	Poland	Adults (clinical)	290	Schizophrenia spectrum disorder	CBT+	Active	Unable to determine	Unable to determine	Unable to determine	0.01	-
Kuhn 2017	PTSD Coach	USA	Adults (subclinical)	120	Trauma-related disorder	Multimodal/other	Passive	Available	Both app stores	Free	0.35	!
Kuhn 2022	Insomnia Coach	USA	Adults (subclinical)	50	Sleep disorder	CBT	Passive	Available	Both app stores	Free	0.14	!
Kulikov 2023	Spark	USA	Adolescents (subclinical)	60	Depressive disorder	CBT	Active	Not available	N/A	N/A	0.20	!
Lacey 2022*	oVRcome	New Zealand	Adults (subclinical)	126	Anxiety disorder	CBT+	Passive	Available	Both app stores	Subscription-Based	-	!
Law 2023	Not determined	Hong Kong	Adults (subclinical)	40	Mixed (transdiagnostic)	Multimodal/other	Passive	Unable to determine	Unable to determine	Unable to determine	−0.90	!
Linardon 2022a	Break Binge Eating	Australia	Adults (subclinical)	392	Feeding/eating/body image disorder	CBT	Passive	Not available	N/A	N/A	0.57	-
Linardon 2022b app 1	Break Binge Eating	Australia	Adults (subclinical)	600	Feeding/eating/body image disorder	Transdiagnostic/Mechanism-Focused	Passive	Not available	N/A	N/A	0.75	-
Linardon 2022b app 2	Breaking the Diet Cycle	Australia	Adults (subclinical)	600	Feeding/eating/body image disorder	Transdiagnostic/Mechanism-Focused	Passive	Not available	N/A	N/A	0.84	-
Liu 2022	General app used to access intervention (e.g. EMA or WhatsApp)	China	Young people (subclinical)	83	Depressive disorder	CBT	Active	Not available	N/A	N/A	1.02	-
LooGee 2021	ExposureTherapy	Australia	Adults (subclinical)	55	Anxiety disorder	Multimodal/other	Passive	Unable to determine	Unable to determine	Unable to determine	−0.65	!
Low 2020	Headspace	Australia	Adults (subclinical)	23	Sleep disorder	Mindfulness-based	Active	Available	Both app stores	Subscription-Based	0.01	!
Lu 2023	Not Determined	China	Adults (subclinical)	145	Mixed (transdiagnostic)	ACT	Passive	Unable to determine	Unable to determine	Unable to determine	−0.05	-
Lu 2023	Good Sleep 365	China	Adults (subclinical)	4052	Sleep disorder	CBT	Active	Available	Apple App Store	Freemium	−0.54	!
Ludtke 2018	Not determined	Germany	Adults (subclinical)	132	Depressive disorder	Multimodal/other	Passive	Not available	N/A	N/A	−0.14	!
Lukas 2021	Mentalis Phoenix (MT-Phoenix)	Germany	Young people (subclinical)	30	Depressive disorder	CBT+	Passive	Available	Both app stores	Free with access code	1.57	!
Lukas 2021	Mentalis Phoenix (MT-Phoenix)	Germany, Austria	Adults (subclinical)	77	Depressive disorder	CBT+	Passive	Available	Both app stores	Free with access code	1.18	!
Mantani 2017	Kokoro-app	Japan	Adults (clinical)	164	Depressive disorder	CBT	Active	Not available	N/A	N/A	0.55	!
Mao 2023	Not Determined	China	Adolescents (subclinical)	30	Anxiety disorder	Multimodal/other	Passive	Unable to determine	Unable to determine	Unable to determine	0.87	!
MazaheriAsadi 2022	Mindfulness Coach Persian Version	Iran	Adults (subclinical)	120	Depressive disorder	Mindfulness-based	Passive	Unable to determine	Unable to determine	Unable to determine	1.73	!
McCloud 2020	Thrive: Feel Stress Free	UK	Adults (subclinical)	168	Mixed (transdiagnostic)	CBT	Passive	Available	Both app stores	Free with access code	0.22	!
McCue 2022	Pathway	USA	Adults (clinical)	40	Depressive disorder	Multimodal/other	Passive	Unable to determine	Unable to determine	Unable to determine	−0.07	!
McKay 2022	A-CHESS	USA	Adults (clinical)	262	Substance use disorder	Multimodal/other	Active and Active	Available	Website	Unclear	−0.65 −0.45	!
McLean 2022*	PTSD Coach	USA	Adults (clinical)	93	Trauma-related disorder	CBT	Passive	Available	Both app stores	Free	-	!
Miner 2016	PTSD Coach	USA	Adults (subclinical)	49	Trauma-related disorder	Multimodal/other	Passive	Available	Both app stores	Free	−0.04	!
Moberg 2019	Pacifica	USA	Adults (subclinical)	500	Mixed (transdiagnostic)	CBT+	Passive	Available	Both app stores	Free with access code	0.52	!
Newman 2021	Lantern	USA	Young people (clinical)	100	Anxiety disorder	CBT+	Passive	Not available	N/A	N/A	0.28	!
Nicol 2022	W-GenZ	USA	Adolescents (clinical)	18	Mixed (transdiagnostic)	CBT	Passive	Unable to determine	Unable to determine	Unable to determine	0.92	!
Niles 2020	Resolving Psychological Stress (RePS)	USA	Adults (subclinical)	1001	Trauma-related disorder	Transdiagnostic/mechanism-focused	Active	Not available	N/A	N/A	.00 .05	!
Oh 2020	Todaki	South Korea	Adults (clinical)	45	Anxiety disorder	CBT+	Active	Available	Google Play Store	Free	−0.01	!
Ponzo 2020	BioBase + paired wearable device (BioBeam)	UK	Young people (subclinical)	262	Anxiety disorder	CBT+	Passive	Unable to determine	Unable to determine	Unable to determine	0.46	!
Price 2022*	CameraHRV	USA	Adults (clinical)	77	Substance use disorder	Relaxation/Stress Management	Active	Available	Both app stores	One time purchase	-	!
RajabiMajd 2020*	CBT for insomnia	Iran	Adults (clinical)	312	Sleep disorder	CBT+	Active	Not available	N/A	N/A	-	!
Roepke 2015	SuperBetter: Mental Health	USA	Adults (subclinical)	283	Depressive disorder	CBT+	Passive	Available	Both app stores	Subscription-Based	0.31	.77!
Rohr 2021	Sanadak	Germany	Adults (subclinical)	133	Trauma-related disorder	Psychoeducation	Active	Unable to determine	Unable to determine	Unable to determine	0.16	!
Roy 2021	Unwinding Anxiety®	USA	Adults (subclinical)	65	Anxiety disorder	Mindfulness-based	Passive	Available	Both app stores	Subscription-Based	1.27	!
Schwob 2023	ImExposure	USA	Young people (clinical)	82	Anxiety disorder	CBT	Active	Not available	N/A	N/A	1.39	!
Seo 2022	Happy Mother	South Korea	Adults (subclinical)	100	Depressive disorder	CBT	Active	Unable to determine	Unable to determine	Unable to determine	0.42	!
Sharma 2023*	Quest	India	Adults (clinical)	30	Substance use disorder	Multimodal/other	Passive	Not available	N/A	N/A	-	!
Shin 2021	Not determined	South Korea	Adults (clinical)	54	Anxiety disorder	CBT	Passive	Unable to determine	Unable to determine	Unable to determine	0.76	-
Soltani 2024	Yara	Iran	Adults (clinical)	64	Depressive disorder	Unclear/not reported	Active	Unable to determine	Unable to determine	Unable to determine	1.32	!
Song 2021	General app used to access intervention (e.g. EMA or WhatsApp)	China	Adults (subclinical)	314	Depressive disorder	CBT	Passive	Not available	N/A	N/A	0.10	+
Stallard 2024	BlueIce	UK	Adolescents (subclinical)	170	Mixed (transdiagnostic)	Multimodal/other	Passive	Available	Apple App Store	Free	0.32	!
Stiede 2022	Keen 2: Track Trichotillomania	USA	Adults (clinical)	15	Conduct/impulse disorder	CBT	Active	Available	Both app stores	Free	0.29	!
Sun 2021	Spirits Healing	China	Adults (subclinical)	168	Depressive disorder	Mindfulness-based	Active	Unable to determine	Unable to determine	Unable to determine	0.48	+
Tan 2023	MoodMission	Malaysia	Adults (subclinical)	48	Mixed (transdiagnostic)	CBT	Passive	Available	Both app stores	Freemium	0.41	-
Teng 2019	General app used to access intervention (e.g. EMA or WhatsApp)	Taiwan	Young people (subclinical)	82	Anxiety disorder	Multimodal/other	Active and Passive	Not available	N/A	N/A	−0.27 −0.05	!
Tighe 2017	ibobbly	Australia	Adults (subclinical)	61	Depressive disorder	ACT	Passive	Available	Both app stores	Free	0.00	!
vanderMeer 2020	SUPPORT Coach	Netherlands	Adults (subclinical)	287	Trauma-related disorder	CBT+	Passive	Available	Both app stores	Free	0.03	!
Versluis 2020*	VGZ Mindfulness Coach	Netherlands	Adults (subclinical)	22	Anxiety disorder	Mindfulness-based	Active	Available	Both app stores	Free	-	!
Werner-Seidler 2023	Sleep Ninja	Australia	Adolescents (subclinical)	164	Sleep disorder	CBT	Active	Available	Both app stores	Free	0.43	!
Wong 2021	Lifestyle Hub	Hong Kong	Adults (subclinical)	79	Depressive disorder	Psychoeducation	Passive	Unable to determine	Unable to determine	Unable to determine	0.64	+
Yang 2017	General app used to access intervention (e.g. EMA or WhatsApp)	China	Young people (subclinical)	76	Anxiety disorder	CBT	Active and Active	Not available	N/A	N/A	.23 .43	!
Zainal 2023	Not determined	USA	Young people (clinical and subclinical)	110	Anxiety disorder	Mindfulness-based	Passive	Not available	N/A	N/A	0.05	+
Zainal 2024	The PACO app	USA	Young people (subclinical)	191	Anxiety disorder	Mindfulness-based	Active	Not available	N/A	N/A	0.41	!
Zhang 2023	Resleep	China	Adults (clinical)	82	Sleep disorder	CBT	Active	Unable to determine	Unable to determine	Unable to determine	0.44	-
Zhang 2023	Not Determined	China	Adults (subclinical)	160	Mixed (transdiagnostic)	Mindfulness-based	Passive	Unable to determine	Unable to determine	Unable to determine	0.55	!
Zhao 2023	Not determined	China	Adults (subclinical)	221	Trauma-related disorder	ACT	Passive	Unable to determine	Unable to determine	Unable to determine	.77 .51	!
Zimmer 2021	Phobys	Switzerland	Young people (clinical and subclinical)	66	Anxiety disorder	Multimodal/other	Passive	Available	Google Play Store	Free	0.54	+

‘*’ studies not included in the meta-analysis. ‘^’Actissist content available in the CareLoop app, which is currently accessible with an access code provided by a clinician. Risk of bias ratings: ‘+’ low risk; ‘!’ = some concerns; ‘-’ = high risk, *N/A* not applicable. *Free* free to service user.

### Control group type

Control groups included both passive control conditions (64 studies; 58%), such as waitlist and no-treatment conditions, and active control conditions (38 studies; 35%), including other digital interventions, psychoeducational content or treatment-as-usual. In 8 studies (7%), more than one control was used, 3 using multiple active controls and 5 combining an active and passive control.

### Conditions targeted

Across studies, the most commonly targeted conditions were depression (31 studies; 28%), followed by anxiety (25 studies; 23%), mixed/transdiagnostic conditions (16 studies; 15%), sleep conditions (9 studies; 8%), trauma related conditions (8 studies; 7%), substance use or eating related conditions (6 studies each; 5%), schizophrenia spectrum conditions (5 studies; 5%), obsessive compulsive conditions (2 studies; 2%), and bipolar (1 study; 1%) and conduct conditions (1 study; 1%).

### Country of origin

The majority of studies were conducted in high income countries (77%), followed by upper middle-income countries (21%), 2% in lower middle-income countries, and none in low-income countries. Geographically, studies were distributed across Europe (*n* = 39; 35%), Asia (*n* = 31; 28%), North America (*n* = 31; 28%), Oceania (*n* = 8; 7%), and Africa (*n* = 1; 1%). The most frequent countries of origin were USA (31 studies), followed by China (11 studies), Germany and UK (8 studies each), Australia and Netherlands (7 studies each), Sweden and South Korea (5 studies each), Iran and Italy (4 studies each), Japan and Hong Kong (3 studies each), Taiwan and India (2 studies each), and single studies in Austria, Ireland, Malaysia, New Zealand, Poland, South Africa, Spain, Switzerland and Turkey, as well as one study which took place in Germany and Austria.

### App characteristics

A summary of app characteristics is provided in Table 1. Across the included studies, 112 interventions were evaluated, with 2 studies including two apps. Across the studies, 81 self-guided smartphone apps were unique, 20 could not be identified by name, and 11 were assessed in multiple (*n* = 13) trials.

The majority of interventions were based on Cognitive Behavioural Therapy (CBT) principles (*n* = 55), followed by mindfulness-based interventions (*n* = 12), and other approaches, including Acceptance and Commitment Therapy (ACT; *n* = 4), behavioural activation (*n* = 4), psychoeducation (*n* = 3), and relaxation/stress management (*n* = 1). Many apps were multimodal interventions combining several different therapy components (*n* = 24) or transdiagnostic interventions targeting specific mechanisms (*n* = 8), and one study did not clearly report a clear therapeutic framework (*n* = 1).

### App availability

Of the 81 unique self-guided mental health apps identified across the included studies, 42 (52%) were publicly available at the time of the review, 18 (22%) were confirmed as unavailable on any public platform, and the availability of 21 (26%) could not be determined because they were not found on the Apple App Store, Google Play Store, or via Google search, and the authors did not respond to availability queries.

### Source of app availability

The availability of apps was examined across Apple and Google Play (Android) App Stores, as well as broader web searches and via author contact. Among the 42 available apps, 33 (79%) were accessible within both Apple and Google Play Stores, 4 (9%) via the Google Play Store, 3 (7%) via the Apple App Store, and 2 (5%) via a public website with download instructions.

### App cost

Cost was determined by reviewing the app’s listing in the Apple App Store, Google Play Store, or official website, including any information on in-app purchases, subscription models, or paywall access. Among the 42 publicly available apps, 17 (40%) were free to download and use, 9 (21%) were free with an access code, 8 (19%) involved a subscription to access full features, 5 (12%) followed a freemium model (free download with in-app purchases), and 2 (5%) required a one-time payment. Cost status could not be determined for 1 available app. Of conditions that had more than two available apps, 30-38% were freely available.

### Publication age and app availability status

The time between publication and app availability was calculated by subtracting the year of study publication from the year of the availability search. Across all studies, the average interval was 4 years (standard deviation = 2.2 years). For studies with available apps, the average interval was 4 years (standard deviation = 2.2 years), while for unavailable apps it was 4.3 years (standard deviation = 2.6 years). This difference was not significant (*p* = 0.73), suggesting that app availability is not influenced by the time elapsed between study publication and the availability search.

### App availability across conditions

Of unique apps whose availability could be determined, availability across different target mental health conditions is shown in Table 2. Overall, availability was generally similarly distributed across conditions, with some notable exceptions. Apps targeting transdiagnostic conditions (11 available; 1 unavailable) and sleep (6 available; 1 unavailable) had the highest proportion of available apps. Further, the few apps targeting obsessive-compulsive disorder and conduct/impulse disorder were all available.

**Table 2:** Number and availability of apps targeting different mental health conditions, where availability can be determined

Target condition	Publicly available	Publicly unavailable	Total availability determined
Depressive disorder	8 (57%)	6 (43%)	14
Anxiety disorder	10 (77%)	3 (23%)	13
Mixed (transdiagnostic)	11 (92%)	1 (8%)	12
Sleep disorder	6 (86%)	1 (14%)	7
Feeding/eating/body image disorder	2 (50%)	2 (50%)	4
Substance use disorder	2 (50%)	2 (50%)	4
Trauma-related disorder	2 (67%)	1 (33%)	3
Schizophrenia spectrum disorder	1 (33%)	2 (67%)	3
Obsessive-compulsive disorder	2 (100%)	0 (0%)	2
Conduct/impulse disorder	1 (100%)	0 (0%)	1

Three apps that targeted different conditions within different studies have been included under both target conditions.

### App availability across regions

Of unique apps whose availability could be determined, availability according to the region the study took place is shown in Table 3. High and upper-middle income countries had approximately the same proportion of availability (72% and 71%, respectively), with no apps publicly available within lower-middle income countries. Where regions had more than 5 apps with determined availability, the greatest proportion were available from Europe, followed by Oceania, North America, and Asia. Each of the two apps evaluated in Africa was available.

**Table 3:** Number and availability of apps whose evidence is generated across different regions, where availability can be determined

Region of app study	Publicly available	Publicly unavailable	Total availability determined
Economic classification			
High income countries	38 (72%)	15 (28%)	53
Upper middle-income countries	5 (71%)	2 (29%)	7
Lower middle-income countries	0 (0%)	1 (100%)	1
Low income countries	NA	NA	NA
Geographic region			
Africa	2 (100%)	0 (0%)	2
Asia	4 (50%)	4 (50%)	8
Europe	18 (86%)	3 (14%)	21
North America	14 (61%)	9 (39%)	23
South America	NA	NA	NA
Oceania	5 (71%)	2 (29%)	7

Two apps were evaluated in different studies in different regions and have been included under both regions.

### Study quality assessment

Study quality for all included studies was assessed using the Cochrane Risk of Bias 2 (RoB 2) tool (see Table 1). Overall, methodological quality was mixed: 10% (*n* = 11) of studies were rated as low risk, 76% (*n* = 84) as having some concerns, and 15% (*n* = 15) as high risk. The most frequent sources of bias were unblinded allocation and outcome measurement, particularly in cases where outcome assessors were not blinded or where outcomes relied solely on self-report from participants aware of their allocation. A further common issue was the absence of pre-registered analyses. In contrast, most studies were judged to be at low risk for bias in randomisation and allocation procedures, deviations from intended interventions, and missing outcome data.

A chi-squared test showed no significant association between app availability (yes vs no) and risk of bias rating (low, high, some concerns) (*χ*² = 0.32, *p* = 0.851).

### Meta analysis: comparison of available vs unavailable apps

Table 4 presents the results from the meta-analysis. The total pooled effect size comparing publicly available apps to control conditions on primary symptom outcomes at post-test was 0.33 (95% CI = 0.19, 0.47), with high heterogeneity (*I*^2^ = 89%) and a broad prediction interval (PI = −0.60, 1.27). The total pooled effect size comparing non-publicly available apps to control conditions on primary symptom outcomes at post-test was 0.45 (95% CI = 0.32, 0.58), with high heterogeneity (*I*^2^ = 81%) and a prediction interval of –0.41 to 1.32. This difference in effect size was not significant (*p* = 0.228).

**Table 4:** Results from the meta-analyses

	Publicly available					Not publicly available				
Analysis	*N*_comp_	*g* (95% CI)	PI	*I*^2^		*N*_comp_	*g* (95% CI)	PI	*I*^2^	*p*
Total effect	52	0.33 (0.19, 0.47)	−0.60, 1.27	89%		54	0.45 (0.32, 0.58)	–0.41, 1.32	81%	0.228
High risk of bias removed	48	0.32 (0.18, 0.47)	−0.61, 1.26	89%		46	0.45 (0.30, 0.60)	−0.61, 1.26	82%	0.241
Outliers removed	46	0.33 (0.19, 0.48)	−0.55, 1.23	89%		41	0.43 (0.33, 0.52)	−0.01, 0.88	53%	0.283
Trim and fill estimate	12 trimmed	0.17 (0.04, 0.30)	-	-		12 trimmed	0.65 (0.49, 0.77)	-	-	-
One effect per study (smallest)	48	0.36 (0.25, 0.46)	−0.25, 0.97	74%		48	0.42 (0.28, 0.56)	−0.46, 1.30	81%	0.488
One effect per study (largest)	48	0.36 (0.20, 0.52)	−0.68, 1.41	90%		48	0.45 (0.30, 0.60)	−0.46, 1.37	82%	0.433
Passive control only	38	0.43 (0.32, 0.55)	−0.13, 1.01	68%		30	0.51 (0.33, 0.69)	−0.37, 1.40	80%	0.492
Active control only	15	0.01 (-0.21, 0.24)	−0.85, 0.89	89%		25	0.38 (0.19, 0.56)	−0.48, 1.24	80%	0.016

*N comp* number of comparisons, *PI* prediction interval, *p* difference in effect size for publicly available vs not publicly available comparisons.

We conducted a series of sensitivity analyses to see if any differences in effect sizes emerged for publicly available compared to non-publicly available apps among different conditions. Effect size for publicly available apps vs. non-publicly available apps did not differ significantly across these sensitivity analyses, with one exception: in the subset of studies that delivered an active control, a significantly (*p* = 0.016) smaller effect size was observed for publicly available apps (*g* = 0.01, 95% CI = −0.21, 0.24) relative to non-publicly available apps (*g* = 0.38, 95% CI = 0.19, 0.56).

## Discussion

To our knowledge, this is the first systematic review and meta-analysis of self-guided mental health apps focused explicitly on translation; that is, understanding the availability of evidence-based resources and the extent to which evidence is translated into real-world deployment. Approximately half (52%) of self-guided mental health apps evaluated in a controlled trial were publicly available at the time of review. According to our meta-analysis, app availability did not significantly differ by effect size (*p* = 0.228). Compared to controls, the pooled effect size was 0.33 for available apps and 0.45 for unavailable apps, with high heterogeneity in both groups. However, effect size did significantly differ (*p* = 0.016) between available (*g* = 0.01) and unavailable (*g* = 0.38) apps when compared to active controls. Strikingly, available apps were not significantly efficacious when compared to active controls. Associations between risk of bias and app availability were not significant, with evidence quality not influencing app availability. A greater proportion of transdiagnostic (92%) and sleep disorder (86%) apps were available, potentially due to their broad applicability in the marketplace.

The findings that strength of effect and study quality are not associated with app availability are novel and indicate that app translation decisions are not solely based on evidence quality or efficacy outcomes. This result is likely influenced by multiple intersecting factors. On the commercial side, it may represent a lack of value placed on evidence by market forces and commercial dynamics. On the research side, it may be indicative of a prior under-prioritisation of dissemination beyond research settings by academic structures, funding agency priorities, and institutional incentives^18^. As a result, academic teams are not always equipped, supported, or incentivised to take digital interventions beyond the research environment. While researchers have the scientific and clinical expertise critical for intervention development and evaluation, they may lack the knowledge, institutional infrastructure, commercial support, or implementation pathways needed to disseminate, sustain, and scale interventions. Together these factors have contributed to a significant gap between effectiveness and access, evidenced by our finding that available apps were not effective when compared to active controls.

Indeed, the surprising finding that unavailable apps demonstrated larger effect sizes than available apps when compared with active control conditions may lend further support to the interpretation that translation decisions are not made on evidence quality alone. Another possible explanation is that available apps may have been more strongly shaped by translation considerations such as usability, engagement, and scalability that may have impacted efficacy. However, given the heterogeneity of interventions and the relatively small number of studies contributing to subgroup analysis, interpretations should be interpreted with caution. What the collective findings do highlight is that research examining the factors and conditions that do influence the successful translation of research-developed mental health apps would shed further light on the nuance of translation in the field.

The identified evidence-availability gap is further supported by the known predominance of evidence-absent apps. Previous literature consistently reports a lack of quality, evidence, and oversight in the app marketplace^9,11–14^, with estimates that a mere 3% of apps have published efficacy^9^. Our results appear to confirm the unprivileged nature of quality evidence in the digital mental health app marketplace, given that our systematic identification of trialled apps surfaced 42 available self-guided apps, a small fraction (~0.5%) of the estimated 10,000 mental health apps available. However, the app marketplace is itself heterogeneous, with digital mental health apps spanning a wide spectrum, from general wellness tools to digital therapeutics subject to regulatory oversight. Thus, when looking at role of evidence in app availability, this variation must be considered.

Although approximately half of included apps were available, with the app stores, unsurprisingly, being the predominant access avenue (79% available in both stores), there is little complementary literature on app dissemination. This contrasts with the growing literature on the implementation of digital mental health innovations within clinical, school, or other settings^2,7,19,20^. Particularly for self-guided app interventions, where direct-to-consumer models are often the predominant translation pathway, literature regarding successful dissemination strategies is a particularly salient research gap, given evidence that availability does not equate to uptake or engagement^21,22^. Indeed, the distinction between availability and discoverability was evidenced by a large proportion (26%) of included apps’ availability unable to be determined. The direct-to-consumer app translation pathway may currently reflect diffusion, the natural, passive spread of information, rather than dissemination, the active, planned effort to spread information to specific audiences^23^. With an increasing emphasis on translation in policy and funding applications, funding structures must also adapt to support the dissemination and visibility of trialled evidence-based apps, which compete for users within commercialised app marketplaces.

Finally, although apps have been proposed as a solution to low mental health accessibility globally, particularly where mental health systems are underdeveloped^24,25^, results observed that only two studies (2%) evaluated self-guided apps in low and lower-middle income countries (LMICs). Self-guided apps that do not rely on other mental health infrastructure for successful use are particularly relevant in this context, but LMIC research remains underrepresented in digital mental health^2,25–27^. These results highlight the importance of collaborating with researchers in LMICs to develop or locally adapt, trial, and disseminate evidence-based resources. These are critical next steps to ensuring technological solutions improve, rather than contribute to, existing inequities.

Although this study provides novel data-driven exploration of app translation in digital mental health, some methodological limitations exist. First, online and app store searches for app availability were performed in high income English-speaking countries. It is possible that apps are accessible within app stores local to where the included app research took place. While we attempted to mitigate this potential bias by contacting authors if searches failed find apps, we only received a 50% response rate. Further, this bias is likely also reduced as most apps (77%) were researched in high income countries (98% in high or upper-middle income countries). Second, this study reports availability at a point in time, however, availability is a dynamic variable. Funding and business models, regulations, resources, and technology advancements are all subject to rapid change and impact continued availability. Thus, the translation of researched apps may be underestimated in the current study, as unavailable apps may have previously been available or may become available. Similarly, available apps may become unavailable. However, results indicated that time since publication did not appear to influence app availability. Further, research has reported that discoverability is also dynamic, as app store search results are unstable, particularly for the Google Play store^28^, also potentially impacting availability results. Finally, mentioned above, availability does not equate to uptake or use of app resources for mental health. Understanding uptake through publicly available data is challenging due to limited reporting of app downloads.

This article demonstrates that just half (52%) of researched are apps available, with 21% freely available, highlighting a structural misalignment between the generation of evidence and its translation into accessible mental health resources. Strikingly, neither evidence strength nor evidence quality were significantly related to app availability, suggesting that current market and commissioning environments do not adequately reward or prioritise empirically supported interventions. Addressing this gap will require coordinated action, from developers, funders, policymakers, marketplaces, and the research community to create clearer pathways for translating validated tools into real-world products. Indeed, there has already been a shift among various funding agencies towards a greater focus on dissemination and implementation. Strengthening these translation mechanisms is essential if evidence-based digital interventions are to achieve their intended public health impact and reach the individuals who stand to benefit most.

## Methods

This review was prospectively registered with the PROSPERO International Prospective Register of Systematic Reviews (registration ID: CRD420251079654), no protocol was prepared, and no amendments to the review were made. The review was conducted in accordance with the Preferred Reporting Items for Systematic Reviews and Meta-Analyses^29^ (PRISMA 2020; see Supplementary Table 1 for checklist).

### Eligibility criteria

Studies were eligible for inclusion if they: (1) were published in a peer-reviewed journal in English between January 2006 and the date of the search; (2) included adults, or young people aged 12 years or older, with a confirmed diagnosis of a DSM or ICD-listed mental health disorder, or with subclinical symptoms assessed using validated psychometric instruments; (3) evaluated a smartphone-based psychological intervention aimed at improving mental health symptoms and/or a transdiagnostic mechanism (e.g., repetitive negative thinking); (4) delivered the intervention in a self-guided format, defined as not requiring active human support for therapeutic benefit - minimal contact (e.g., check-ins) was permitted if it was not a core therapeutic component; (5) employed a controlled study design in which a group receiving the intervention was compared to a control group not exposed to the intervention (e.g., waitlist, treatment-as-usual, or active comparator); and (6) reported post-treatment means and standard deviations (or equivalent) for primary outcomes related to mental health symptoms or transdiagnostic mechanisms.

Studies were excluded if they: (1) were case studies, reviews, theses, book chapters, or conference abstracts; (2) reported duplicate data published elsewhere; (3) were not published in a peer-reviewed journal; (4) were not available in English; (5) primarily focused on physical health conditions with secondary mental health outcomes (e.g., diabetes, HIV); (6) involved interventions focused solely on symptom monitoring or data collection without therapeutic intent; or (7) evaluated interventions not delivered via a smartphone platform.

### Search strategy

A systematic search was conducted on 11 July 2024, across four electronic databases: PsycINFO, MEDLINE, Web of Science, and the Cochrane Central Register of Controlled Trials (CENTRAL). The search covered publications from January 2006 to 11 July 2024 and was limited to peer-reviewed articles published in English. The search strategy combined terms related to mental health, digital interventions, mobile applications, and trial design (see Supplementary for Syntax). Reference lists of included studies were screened for additional eligible trials.

### Study Selection

Search results were imported into Covidence systematic review software. After removing duplicates, titles and abstracts were screened by a single reviewer against inclusion and exclusion criteria (Stage 1). Full texts of potentially eligible studies were then independently reviewed by two reviewers against inclusion/exclusion criteria (Stage 2). Discrepancies were resolved through discussion and consensus.

### Data extraction

Data extraction was completed by one reviewer and checked by a second reviewer for at least 50% of the included studies. Extracted data included: 1) Study characteristics (e.g., authorship, publication year, country); 2) Sample demographics, including age categorised as young people (aged 12–25) or adults (aged 26 + ) aligned with early intervention research^30^, and clinical characteristics; 3) App name, target condition, intervention type, and country of origin; 4) Control group details; 5) App availability and cost at the time of review; 6) Outcome data (pre/post means, standard deviations, sample sizes); 7) Risk of bias assessments.

For studies with missing or unclear data, attempts were made to contact the corresponding authors.

### App availability search strategy

Following data extraction, between July 9 and August 13 2025, a secondary search was conducted to determine the public availability of each unique self-guided app evaluated in the included studies. Each app was first searched by name in the Apple App Store and Google Play Store. If not located, a Google search (top 10 pages) was conducted to identify a website with direct download instructions. If availability was still unclear, corresponding authors were contacted for clarification. All app searches were completed by one reviewer and checked by a second reviewer.

App availability was defined as being publicly accessible for download via app stores, a website with clear instructions, or another publicly accessible route as confirmed by the study authors.

### Risk of bias assessment

Risk of bias for each included study was independently assessed by two reviewers using the Cochrane Risk of Bias 2.0 tool^31^, evaluating five domains: randomisation process, deviations from intended interventions, missing outcome data, outcome measurement, and selection of the reported result. Disagreements were resolved by consensus or consultation with a third reviewer.

### Statistical analysis

Meta-analyses were conducted using Comprehensive Meta-Analysis Version 4^32^. For each comparison between the intervention and control, the effect size was calculated by dividing the difference between the group means on the primary symptom measure at post-test by the pooled standard deviation. If only standard errors or 95% confidence intervals were reported, these were manually converted to standard deviations. Effect sizes were then reported as Hedges’ *g* to correct for small sample bias^33^. To calculate a pooled effect size, each study’s effect size was weighted by its inverse variance. For all outcomes, a positive *g* indicates that the intervention group fared better than the control group. Effect sizes of 0.8 were interpreted as large, while effect sizes of 0.5 as moderate, and effect sizes of 0.2 as small. The 95% prediction interval was also computed to estimate the range within which individual future observations are expected to fall.

We conducted a series of sensitivity analyses to assess whether the main findings were robust. First, we re-calculated pooled effects after excluding trials that received a high risk of bias rating. Second, we recalculated pooled effects while excluding outliers, in which a study is defined as an outlier when the 95% CI of the effect size does not overlap with the 95% CI of the pooled effect size. Third, we calculated the pooled effect when applying the trim-and-fill procedure that corrects for small-study bias^34^. Fourth, if a trial had more than one intervention or control arms, we re-calculated pooled effects when excluding the smallest and largest effect per comparison (to maintain statistical independence). Fifth, we recalculated pooled effects for both passive and active controls, considering evidence showing that control group type can strongly influence effect size estimates^35^.

Random effects models were used for all analyses. Heterogeneity was examined by calculating the I2 statistic, which quantifies heterogeneity revealed by the Q statistic and reports how much overall variance (0–100%) is attributed to between-study variance^36^. All pooled effects were stratified on the basis of the public availability of the app and whether the effect size varied significantly was examined under mixed effects models.

## Supplementary informations

Supplementary information

## Data Availability

The datasets analysed during this review are available from the corresponding author upon request.

## Code availability

The code used for the analyses in this review is available from the corresponding author upon request.
